# Spinal Cord Injury Management Based on Microglia-Targeting Therapies

**DOI:** 10.3390/jcm13102773

**Published:** 2024-05-08

**Authors:** Thomas Gabriel Schreiner, Oliver Daniel Schreiner, Romeo Cristian Ciobanu

**Affiliations:** 1 Department of Medical Specialties III, Faculty of Medicine, University of Medicine and Pharmacy “Grigore T. Popa”, 700115 Iasi, Romania; schreiner.thomasgabriel@yahoo.com; 2 First Neurology Clinic, “Prof. Dr. N. Oblu” Clinical Emergency Hospital, 700309 Iasi, Romania; 3 Department of Electrical Measurements and Materials, Faculty of Electrical Engineering and Information Technology, Gheorghe Asachi Technical University of Iasi, 700050 Iasi, Romania; oliver090598@yahoo.com; 4 Medical Oncology Department, Regional Institute of Oncology, 700483 Iasi, Romania

**Keywords:** microglia, neuroinflammation, spinal cord injury, blood-spinal cord barrier, microRNA, stem cells, cytokines

## Abstract

Spinal cord injury is a complicated medical condition both from the clinician’s point of view in terms of management and from the patient’s perspective in terms of unsatisfactory recovery. Depending on the severity, this disorder can be devastating despite the rapid and appropriate use of modern imaging techniques and convenient surgical spinal cord decompression and stabilization. In this context, there is a mandatory need for novel adjunctive therapeutic approaches to classical treatments to improve rehabilitation chances and clinical outcomes. This review offers a new and original perspective on therapies targeting the microglia, one of the most relevant immune cells implicated in spinal cord disorders. The first part of the manuscript reviews the anatomical and pathophysiological importance of the blood-spinal cord barrier components, including the role of microglia in post-acute neuroinflammation. Subsequently, the authors present the emerging therapies based on microglia modulation, such as cytokines modulators, stem cell, microRNA, and nanoparticle-based treatments that could positively impact spinal cord injury management. Finally, future perspectives and challenges are also highlighted based on the ongoing clinical trials related to medications targeting microglia.

## 1. Introduction

Spinal cord injuries (SCIs) are extremely stressful experiences for patients, frequently leaving them with debilitating and lifelong neurologic impairments. Numerous short-term and long-term consequences, such as loss of motor and sensory function, failure of autonomic function, and an elevated risk of morbidity and mortality, are regularly associated [1]. Based on the most recent epidemiological data, the incidence of traumatic SCI is around 54 cases per one million people in the United States alone, making it over 15,000 new cases yearly [2]. These figures are a constant addition to the already nearly half a million Americans permanently incapacitated after SCI [3]. The numbers have increased even more when considering the worldwide situation, with variable prevalence according to region and country [4]. In most developed countries, the average incidences were between 20 and 50 per million annually [5].

Regarding the etiology of SCI, the leading cause remains vehicle crashes/road accidents, followed by falls, and secondary to violent acts [6]. Sport-related injuries and post-surgical trauma represent only a minority of the group. Another relevant aspect of SCI is related to underlying mechanisms, direct injury, transient or persistent compression, and laceration being the main possibilities [7]. Subsequently, the degree of injury can be described as complete or incomplete, or more specifically via different scale systems, such as the Frankel scale or the American Spinal Injury Association (ASIA) scoring system [8]. These classifications are relevant for acute setting treatment and chronic phase rehabilitation. The severity of SCI is directly correlated with the long-term prognosis, and despite the use of several therapeutic measures that might be helpful in minor lesions, some severe traumatic injuries have unsatisfactory outcomes.

Since young, healthy persons are more likely to suffer traumatic SCI, the resulting loss in quality-adjusted life years and economic damages can be enormous [9]. SCI imposes a significant financial strain on the acute care environment and the longer-term rehabilitation that frequently follows the initial injury [10]. The magnitude of the spinal cord damage, the level of disability, and the individual variables of each case (comorbidities, lifestyle) significantly impact the costs. Strictly discussing figures, according to a recent systematic review [9], the direct costs related to SCI are very variable, with the mean costs of acute care ranging from a couple hundred to over $600,000. Rehabilitation costs exceed the acute treatment expenses, highlighting the long-term financial burden of the disease and the importance of improving acute setting treatment. Besides the direct costs, indirect expenses should also be considered. Residual neurologic impairment may significantly affect social and professional reinsertion, negatively impacting employment status and earnings. In this regard, a recent observational study showed that SCI is correlated with a decreased employment rate, leading to severe lifetime indirect costs for injured persons [11].

Considering the significant individual and socioeconomic burden of SCI in both acute and chronic settings, major improvements in the therapeutic approach are crucial. Along with surgical intervention, adjuvant drug treatment could be a promising avenue to increase patients’ general outcomes and quality of life. Given the extensive fresh knowledge of the role of the blood-spinal cord barrier (BSCB) in spinal cord disorders, this review aims first to investigate the microglial cell role in spinal cord injury. Neuroinflammation and BSCB structural damage are two key events explaining the severity of SCI, and modulating them is a promising tactic to improve clinical outcomes. Thus, the central part of the article comprises the most relevant targeted therapies aiming to modulate microglia, subsequently reducing neuroinflammation, promoting neural repair, and sustaining clinical rehabilitation. Finally, with many ongoing clinical trials and a high possibility of new drugs being approved in the next few years, future perspectives and challenges are also discussed.

## 2. The Blood-Spinal Cord Barrier in Physiologic and Traumatic Conditions

**Figure 1:**
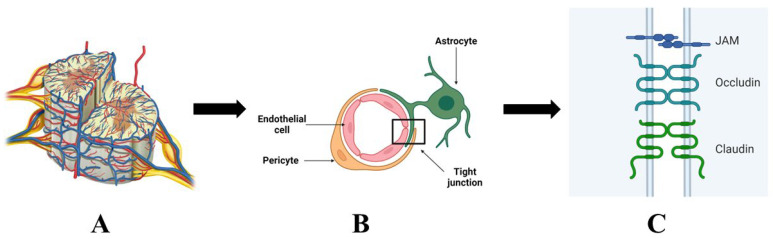
The blood-spinal cord barrier in physiological conditions: (**A**) blood supply of the spinal cord; (**B**) schematic representation of the blood-spinal cord barrier; (**C**) schematic representation of a tight junction (designed by using Biorender.com, accessed on 23 March 2024).

The BSCB is a unique structure in the human body that acts as a specialized interface between the bloodstream and the spinal cord (see Figure 1). Physiologically, it is a highly selective barrier that regulates the bidirectional flow of ions, nutrients, lipids, and other small molecules [12]. The BSCB can also be considered the functional equivalent of the blood-brain barrier (BBB) at a lower central nervous system (CNS) level. While the BBB has been extensively studied in several pathological conditions, including traumatic [13] and neurodegenerative disorders [14], the BSCB was not until recently in-depth investigated, being considered for a long time only a natural extension of the BBB at the spinal cord level. However, this paradigm has changed, with recent research showing notable structural and functional differences between the two entities. The spinal cord is a far more flexible organ than the brain, with immune responses located in the spinal cord having distinct clinical characteristics and necessitating appropriate intervention. Thus, SCI leads to more than simple mechanical damage to the BSCB and the neighboring tissues, triggering many molecular and cellular events that need better understanding [15]. With significant scientific advancements on the topic, an updated view of BSCB’s structure is further detailed.

The BSCB comprises cellular and non-cellular components, with endothelial cells being one of the principal cells sustaining BSCB’s impermeability. This endothelial tissue has unique characteristics, limiting the trans-flow of potential neurotoxic substances from the bloodstream to the spinal cord and serving as the principal molecular border at the spinal cord level [16]. To ensure high selectivity, the endothelial cells have a small number of endocytic processes and are closely sealed by tight junctions (TJs) [17], special intercellular protein bridges that will be discussed in detail below. Another particularity of the BSCB’s endothelium is the higher concentration of mitochondria compared to the BBB’s endothelium, which might be explained by the increased metabolic need in the spinal cord [18].

The TJs, intercellular structures that ensure the tightness of BSCB, are of particular interest. Several types of proteins form the TJ, divided into three classes: claudins (over 25 member proteins, with claudin-1 and claudin-5 the most relevant ones for BSCB), MARVEL proteins (occludins), and the Immunoglobulin superfamily membrane proteins, such as JAM-A/-B/-C, and the endothelial cell-selective adhesion molecule [19]. The primary constituents of the BSCB’s TJs are claudin-1, claudin-5, occludin, and Zonula occludens (ZO-1) [20]. Moreover, BSCB’s higher permeability than BBB is thought to result from the lower levels of occludin and ZO-1 in its structure [21].

Other cells of the BSCB complex, such as pericytes and astrocytes, are also important in modulating the endothelium’s particular phenotype. Pericytes are essential in sealing the small vessel walls but also influence TJ formation and endothelial cell migration, differentiation, and proliferation [22]. Pericytes may also direct the blood flow, particularly the ones expressing muscle cell phenotype [23]. The heterogeneity of the pericyte population is worth mentioning, with several markers such as aminopeptidase-N and the platelet-derived growth factor receptor most frequently used in molecular studies. Moreover, the versatility of pericytes may also be related to the spinal cord region, as their functionality is dependent on the location and spinal cord anatomy, having therapeutic implications. Compared to the BBB, the BSCB has a lower number of pericytes, which additionally explains its increased permeability.

Glial cells, particularly astrocytes, may play a significant role in ensuring the structural and functional well-being of the BSCB, considering their high number in the spinal cord. The astrocytic feet have a critical position, being in close contact with neurons, pericytes, and endothelial cells [24]. The supportive feature of astrocytes is based on the secretion of a variety of chemical mediators, including TGF-β, glial-derived neurotrophic factor (GDNF), basic fibroblast growth factor (bFGF), and angiopoietin-1 (Ang1) [25]. By expressing aquaporin-4, perivascular astrocyte endfeet wrap around endothelial cells and aid in controlling brain water transport [26]. Additionally, astrocytes have the ability to control the production and location of several transport proteins and endothelium-specific enzyme systems. Astrocytes’ close interplay with the endothelial cells ensures the barrier qualities by sustaining the existence of pumps and specialized ion channels, structures that are essential in maintaining transendothelial electrical resistance [27]. On the other hand, endothelin produced by the endothelial cells modulated astrocyte growth and differentiation by stimulating the production of nerve growth factors such as brain-derived neurotrophic factor (BDNF) [28]. Considering the functional anatomy of the spinal cord, the close interplay between neurons and astrocytes might also impact the astrocyte phenotype, with slight differences among the astrocytes located in the anterior and posterior horns and columns. Despite not being completely understood, these differences may have therapeutic consequences and are relevant for the optimal drug choice.

Besides the cellular components, non-cellular parts are also of interest for BSCB’s structure. The basal lamina is a relevant arrangement of laminin, collagen, fibronectin, and proteoglycans situated on the abluminal surface of the endothelial cells [29]. Its leading role is to prevent the entry of macromolecules by separating pericytes from endothelial cells.

From the functional point of view, BSCB’s principal properties are similar to the BBB’s features. The BSCB, as its name implies, functions as a physical barrier to various blood-borne molecules that may be advantageous to other organs but could be very harmful to the delicate spinal cord tissue [30]. BSCB is more than a physical border; it is also restrictive when considering polarity and molecular size, explaining the protective role of restricting the entry of various drugs into the CNS. BSCB’s integrity is also essential for maintaining ion homeostasis at the spinal cord level. Specialized ion channels and pumps are of great importance for keeping constant ion and electrolyte concentration, ensuring the appropriate neuronal metabolism and cell functioning [31]. Finally, BSCB is also significantly involved in eliminating metabolic end-products and toxins from the spinal cord. These toxic compounds cross the BSCB, are released into the bloodstream, and are subsequently eliminated through the kidneys or the digestive tract [32].

In the case of spinal cord injury, the BSCB is one of the most vulnerable structures that suffer structural and functional changes. It has been suggested that the activation of matrix metalloproteases (MMPs) is the cause of BSCB dysfunction in SCI [33]. MMPs are involved in a vast number of physiological processes, including inflammation, wound healing, angiogenesis, tissue morphogenesis, and cell migration [34]. Nevertheless, their function also includes breaking down different extracellular matrix (ECM) components, which allows immune cells such as leukocytes and microglia to penetrate barriers like the BSCB [35].

There are several types of MMPs, each being modulated by different substrates and influencing, in turn, various signaling pathways. For example, histone H3K27 demethylase Jmjd3 is up-regulated in response to MMP-3 and -9, resulting in the loss of TJ proteins and increased BSCB permeability [36]. MMP-8 and MMP-12 have demonstrated similar changes in TJ protein abundance and barrier permeability, being encountered as late effects in SCI [37]. The mechanical stress caused by trauma harms the BSCB’s components, such as the endothelial cells, and promotes the development of cation channels. Other cells are also involved, such as astrocytes, which change their phenotype in spinal cord trauma, becoming reactive cells [38]. Calmodulin enhances the up-regulation of aquaporin 4 channels (AQP4), which subsequently explains the increased permeability of the barrier [39]. Cells not belonging to the BSCB in physiological conditions are present in pathological states, including after traumatic injuries. A relevant example is related to macrophages, which are gathered at the BSCB milieu by cytokines and upregulation of perforin [40]. Other inflammatory cells, such as neutrophils, are also observed, closely related to the activity of MMP-3 and the NF-κB signaling [41]. Finally, microglia are also key players in regulating inflammatory changes post-SCI, with their multiple implications detailed further on.

## 3. Microglia in the Context of Spinal Cord Injury

Microglia are the only resident phagocytes in the parenchyma in physiological conditions compared to other border-associated macrophages. The distinction can be difficult, as it is made by analyzing cell surface markers, such as a lower expression of CD45 [42]. Microglia maintain a homeostatic phenotype in the uninjured spinal cord and are involved in several processes, from CNS development to immune surveillance in adults. From the embryological perspective, microglia participate in regulating cerebrovascular development, being present in the developing CNS before the migration of endothelial cells [43]. Microglia play a pivotal role in the generation of the cellular and humoral inflammatory response, along with the subsequent cascade of associated events. There is insufficient explanation for the relationship between microglial cells and the other cells located at the BSCB level and their implication in BSCB regulation.

In traumatic conditions, microglia, astrocytes, and other immune cells get activated. Among the many structures involved in SCI, microglia seem to play an increasing role in the posttraumatic phenomena, particularly in neuroinflammation [44]. When activated, these cells produce inflammatory cytokines (TNF-α, IL-1), chemokines, prostaglandins, and reactive oxygen species. They also suffer morphological changes, such as losing lengthy processes [45]. Microglial cells were thought to change into the M1 phenotype (pro-inflammatory) or the M2 microglia (anti-inflammatory phenotype) by releasing chemokines that aid in tissue repair and vascular endothelial growth factor; however, more recent studies questioned this classification, pointing to a fragile line separating the two states [46].

After injury, phagocytosis of cell debris is crucial for recovery. Microglia initially show low CD68 expression but increase it by the following few days, indicating heightened phagocytic activity [47]. Starting two weeks post-injury, CD68 expression decreases while purinergic receptor P2ry12 increases, suggesting a return to homeostasis. Microglia interact with injured axons from the early phases, while macrophages phagocytose debris much later. Still, microglia show more efficient debris processing than macrophages [48].

Activated microglia exhibit various phenotypes with neurotoxic or neuroprotective effects depending on external stimuli. They contribute to forming a glial reparatory tissue near the injured tissue, aiding in debris clearing and wound compaction. Microglia play a significant role in astrocyte proliferation and induce astrocytic conglomerate formation through cytokine secretion [49]. However, the impact of microglia on astrocytes is not limited to only the induction of astrocyte proliferation; based on microglial phenotype, astrocytes can be activated and change their characteristics, as is detailed further on.

Plexin-B2, a transmembrane receptor that participates in axon guidance and cell migration, is important in injured microglia, as it helps maintain the astrocyte barrier and wound compaction [50]. Glial scar formation inhibits neuronal regeneration post-SCI due to cytotoxic cytokines like chondroitin sulfate proteoglycans (CSPGs), though some CSPGs promote axonal growth.

In the spinal cord, microglia have multifaceted effects on various cells, including neurons. In their resting state, they surveil the immune system, maintain environmental stability, and regulate neural circuit development in the immature CNS. After SCI, microglia phagocytose axon fragments and secrete pro/anti-inflammatory molecules, influencing neuron regeneration. Persistent microglia activation post-injury contributes to neurodegeneration and neurological deficits. Microglia-derived scars can isolate axons from cytotoxic immune cells in core lesions, with their deletion proving disadvantageous to neuron regeneration. Fibroblast growth factor 10 (FGF10) from neurons and microglia/macrophages increases after SCI, aiding in tissue repair and functional recovery via the FGFR2/PI3K/Akt pathway [51].

Research reveals inconsistent findings about the impact of microglia elimination on neuronal repair after injury. Sustained microglia depletion has a minor effect on neurogenesis post-CNS injury, but turnover to a neuroprotective phenotype stimulates functional neurogenesis after traumatic brain injury (TBI) [52]. Short-term depletion of activated microglia followed by regeneration leads to long-term recovery of neurological function and reduction in neurodegenerative processes. Microglia influence neuron regeneration based on their phenotype and action time, with further investigation needed on their effects in different stages.

The relationship between microglia and neurons extends to physical interactions, with microglia modulating neurotransmission and potentially enhancing excitatory neurotransmission. The contact between microglia and damaged axons may lead to phagocytosis and might enhance regeneration through physical contact, challenging the current understanding of neuroinflammation and suggesting new avenues for axonal injury treatments. Specialized nanoarchitecture and purinergic signaling at microglia-neuron junctions potentially surveil and protect neuronal function [53].

Microglia significantly influence non-neuronal cells in the spinal cord, particularly astrocytes, which are closely associated. Microglia rapidly modulate astrocytes’ activity and proliferation in response to environmental changes. After SCI, microglia stimulate astrocyte proliferation and activation, leading to diverse outcomes. They induce the formation of neurotoxic A1 astrocytes through the secretion of interleukine-1α (IL-1α), tumor necrosis factor (TNF), and complement component 1q (C1q) [54]. Even partial microglia presence can activate A1 astrocytes, highlighting their crucial role. Although IL-1α, TNF, and C1q are essential inducers of A1 astrocytes, their depletion alone cannot reverse the astrocyte phenomenon [55].

On the contrary, neuroprotective A2 astrocytes are induced by microglia through fibroblast growth factors (FGF) signaling activation, supporting neuron growth. Astrocytes, in turn, enhance microglial activation and TNF-α production in brain inflammation by producing proinflammatory molecules [56]. Microglia also play a role in astrocyte necroptosis via TLR/MyD88 signaling, impacting SCI secondary damage. Glial scar formation involving microglia and astrocytes can mitigate neuron degeneration post-SCI. Understanding the intricate interactions between microglia and astrocytes and their effects on BSCB repair is crucial for future research in animal models of SCI and for developing effective therapeutic strategies.

Microglia play a pivotal role in influencing the structural and permeability characteristics of BSCB through their close interaction with endothelial cells. In abnormal conditions, microglia increase barrier permeability, promote leukocyte infiltration, and induce angiogenesis. Reactive oxygen species released by activated microglia produce oxidative damage to endothelial cells along with the upregulation in nitric oxide (NO) and inducible nitric oxide synthase (iNOS). Exacerbated by IL-1β and TNF-α, microglia facilitate peripheral leukocyte infiltration into the CNS parenchyma [57]. Moreover, under high glucose conditions, microglia-derived IL-6 activates signal transducer and activator of transcription 3 (STAT3) in endothelial cells, resulting in increased endothelial permeability by downregulating occludin and ZO-1 production in tight junctions [58]. Microglia-derived inflammatory cytokines can degrade extracellular matrix (ECM) proteins and disrupt the BSCB by producing matrix metalloproteinase. Another molecular mechanism involves hypoxia-induced microglia upregulating basigin-2 expression and releasing insulin-like growth factor 1 (IGF-1) via promotion of the phosphatidylinositol 3-kinase (PI3K)-Akt pathway, thereby inducing angiogenesis [59]. Activated microglia, demonstrated in co-culture experiments, promote angiogenesis and migration of retinal microvascular endothelial cells while reducing the production of tight junction proteins by increasing the expression of platelet-derived growth factor-BB and vascular endothelial growth factor-A [60]. In stroke mice, treatment with metformin increases anti-inflammatory cytokine secretion from microglia, facilitating angiogenesis and neurogenesis, ultimately promoting locomotor recovery [61]. Anti-inflammatory cytokines from microglia may aid recovery from ischemic stroke by accelerating angiogenesis by releasing higher amounts of exosomes containing miRNA-26a [62]. In summary, while microglia-derived inflammatory cytokines increase blood vessel permeability, anti-inflammatory molecules contribute to angiogenesis through various signaling pathways.

Finally, microglia modulates the behavior of other relevant cells in the spinal cord, such as oligodendrocytes and progenitor cells. Microglia are crucial in regulating oligodendrocyte survival and function throughout CNS development, aiding normal myelinogenesis and maintaining oligodendrocyte progenitor cells (OPCs) during adulthood in healthy individuals [63]. After SCI, axon demyelination and oligodendrocyte cell death occur, contributing to secondary injuries and persistent neurodegeneration. Microglia exhibit either beneficial or harmful effects on oligodendrocytes in different pathological contexts. Activated microglia can promote oligodendrocyte cell death and phagocytosis through secretion of TNF, NO, and complement, while S100A8/A9-induced microglia activation facilitates OPC apoptosis via the NF-κB signaling pathway [64]. Conversely, the antidepressant fluoxetine decreases oligodendrocyte cell death post-injury by inhibiting microglia activation [65].

Moreover, the transformation of microglia from a proinflammatory to an anti-inflammatory state coincides with oligodendrocyte remyelination initiation. Anti-inflammatory microglia prevent OPC apoptosis and enhance OPC differentiation to oligodendrocytes in vitro, with impaired differentiation following anti-inflammatory microglia depletion [66]. Microglia also facilitate oligodendrogenesis post-CNS injury, with various microglial profiles having diverse impacts on oligodendrocytes and OPCs, necessitating further elucidation of the specific mechanisms [67].

In CNS neurogenesis, microglia stimulated by lipopolysaccharide (LPS) or interferon-gamma (IFN-γ) release cytokines that reduce neural stem/progenitor cell (NSPC) proliferation [68]. However, non-inflammatory microglia support NSPC propagation by producing neurotrophic molecules, while a deactivated microglia-conditioned medium positively affects NSPC proliferation [69]. Additionally, microglial subtypes have distinct impacts on NSPC regulation, with anti-inflammatory molecules supporting oligodendrogenesis and inflammatory molecules promoting neurogenesis. Further research is needed to fully understand the effects of various microglia on NSPCs and the exact underlying mechanisms.

Table 1 summarizes the most relevant cellular and molecular processes modulated by microglia in spinal cord trauma.

## 4. Targeted Therapies Modulating Microglia

Considering the crucial role played by microglia in the hyperacute setting of SCI and their vast influence on the other cellular components of the BSCB and spinal cord nervous tissue, microglia become a valid therapeutic target. Up to the present, several experimental treatments have been proposed, given the double-edged sword role played by microglia in acute trauma, mainly to control secondary neuroinflammation. In instances of neuroinflammation, microglia have the potential to contribute positively to spinal cord repair following injury. However, they can also transition to a destructive role, releasing excessive cytotoxic cytokines and reactive oxygen mediators. Consequently, controlling the quantity or characteristics of activated microglia and reducing inflammation could offer a viable approach to treating SCI and enhancing functional recovery. The optimal timing of microglia-targeting treatments remains controversial, with microglia changing its phenotype possibly multiple times in the post-SCI phases. Additionally, the effects in time of the proposed therapies are also little known, for most of them being difficult to achieve precise timing control. Table 2 summarizes the most relevant therapeutic trials conducted in recent years, highlighting the main directions of possible treatments.

One possible microglia-targeting treatment is based on the use of CSF1R inhibitors. Colony-stimulating factor 1 (CSF1) regulates microglial survival, propagation, and differentiation, making this class of drugs effective in removing microglia [70]. Studies using GW2580, a CSF1R inhibitor, demonstrated reduced proliferating microglia at the injury site in SCI models, leading to improved locomotor recovery [71]. Combining PLX3397, another CSF1R inhibitor, with hydrogel transplantation effectively depleted activated microglia/macrophages, enhancing neural stem/progenitor cell (NSPC) differentiation into neurons and improving functional recovery [72]. However, selective microglia depletion can result in diffuse inflammation and increased lesion size, suggesting the importance of timing in this strategy.

With cytokines as the small polypeptides that influence microglia growth, differentiation, and activation, cytokine therapy is another promising therapeutic option [73]. Cytokine therapy involves both replacement and blocking strategies. IL-4 administration increased anti-inflammatory microglia and macrophages, reducing tissue injury and improving motor outcomes in SCI [74]. FGF1 injection inhibited microglia/macrophage proliferation and activation via the TLR4/NF-κB pathway, promoting injury recovery [75]. Neuregulin-1 (Nrg-1) attenuated microglial response under stress conditions by reducing proinflammatory mediator generation [76]. Nerve growth factor (NGF) enhanced microglial phagocytosis of Aβ and maintained neuronal integrity by inducing anti-inflammatory molecule secretion [77]. Cytokines alter microglial status to improve SCI recovery, but further exploration of cytokines and enhanced drug delivery methods are needed.

Stem cell transplantation, particularly involving human neural stem cells (hNSCs) and adipose mesenchymal stem cells (MSCs), holds promise for treating neurodegenerative disorders and SCI. In models of Alzheimer’s disease and cortical impact injury, hNSC transplantation improved cognitive ability and reduced microglial activation, potentially through the secretion of anti-inflammatory molecules [78]. NSCs are beneficial for neuronal regeneration by modulating microglial secretion of anti-inflammatory cytokines and inhibiting microglial proliferation, migration, and phagocytosis by releasing vascular endothelial growth factor (VEGF) [79]. Similarly, MSCs exert immunosuppressive effects on microglial activation and promote the secretion of anti-inflammatory cytokines [80]. Additionally, NSCs have been shown to enhance damage repair by engulfing anti-inflammatory nanoparticles [81]. Future research may explore novel materials and targeted delivery methods to optimize the therapeutic potential of exogenous NSCs in SCI treatment.

Stem cell-derived extracellular vesicles (EVs), including microvesicles (MVs) and exosomes, have emerged as crucial mediators of paracrine signaling in promoting CNS development and repair. Studies have shown that neural stem cell-derived MVs (NSC-MVs) can reduce microglial activation, alleviate SCI-related impairments, and encourage locomotor recovery by inducing autophagy [82]. MSC-derived MVs also exhibit regulatory effects on microglial activation, lowering proinflammatory cytokine secretion and promoting the expression of anti-inflammatory markers [83]. Additionally, MSC-derived exosomes exert anti-inflammatory effects on microglia by interfering with TLR4 signaling and modulating the expression of microRNAs targeting microglia [84]. The structural differences between BSCB and the BBB facilitate the delivery of EVs, making these stem cell-derived EVs hold promise as cell-free therapies for treating severe damage in SCI, with future research focusing on optimizing their efficacy through the use of new materials and delivery methods.

MicroRNAs (miRNAs) play crucial roles in regulating cellular processes and can modulate microglial function and neuroinflammation [85]. miRNA therapy offers a promising avenue for SCI treatment by targeting inflammatory pathways and promoting anti-inflammatory cytokine secretion. Examples include miR-873a-5p, which inhibits inflammation and enhances anti-inflammatory cytokine release, and miR-133b and miR-124, which reduce proinflammatory factors and inflammation in neurodegenerative conditions [86]. Other miRNAs, such as miR-100, miR-183, miR-23b, miR-34a, miR-150, miR-27a, miR-340-5p, miR-193a, miR-429, and miR-223-5p, also show potential in attenuating inflammation, oxidative stress, and apoptosis post-SCI [87]. These findings highlight the therapeutic potential of miRNA modulation in SCI treatment.

Additionally, recent research has highlighted the significance of the gut-brain axis in CNS degeneration, with the gut microbiota exerting notable effects on microglia function in CNS disorders [88]. Studies using various mouse models, including those with limited bacterial colonization or complete microbiome depletion, have demonstrated distinct microglial abnormalities, such as immature phenotype and altered cellular morphology [89]. Fecal microbiota transplantation has shown promise in reducing microglial activation and providing neuroprotection in conditions like Parkinson’s disease, potentially via modulation of the TLR4/TBK1/NF-κB/TNF-α signaling pathway [90]. Additionally, microbial-derived tryptophan has been implicated in regulating microglial inflammation through the aryl hydrocarbon receptor-mediated production of TGF-α and VEGF-b [91]. While the precise roles of different microbial species and their mechanisms remain unclear, dietary interventions and antibiotic treatments have been shown to influence the gut-brain inflammation axis after SCI. Investigating the interplay between specific microbial strains and microglia holds promise for developing novel therapeutic strategies to mitigate neuroinflammation and SCI in humans.

Finally, while not directly targeting the microglia, several studies have questioned the role of various neuroprotective agents in attenuating BSCB disruption in SCI, indirectly modulating the microglial activity. These agents include neurotrophins, peptide hormones, antioxidants, and bradykinin antagonists, which have significantly reduced protein tracer extravasation, indicating BSCB restoration [92]. Modulation of SUR1/TrpM4 and MMP-9 expression using compounds like ghrelin, 17β-estradiol, protocatechuic acid, and flufenamic acid has been effective in restoring BSCB integrity by regulating neutrophil and macrophage/microglia infiltration [93]. Moreover, the blood supply in close connection to the BSCB plays a role in facilitating the recruitment of monocyte-derived macrophages post SCI. Studies have highlighted the potential modulation of IFN-γ/IFN-γR expression as a novel treatment approach to boost anti-inflammatory molecule recruitment to the injury site [94]. Acidic compounds such as valproic, salvianolic, and oleanolic acids have been used to modulate pathways like STAT1-NF-κB, reducing pro-inflammatory responses and restoring BSCB permeability [95].

**Table 2:** Microglia-targeting therapies in spinal cord injury.

Proposed Therapy	Relevant Drugs/Trials	Microglia Implications	Current Status	References
CSF1R inhibitors	GW2580 PLX3397	Reduction in microglia proliferation Depletion of activated microglia	While selective microglia depletion leads to improved locomotor recovery, it can sustain inflammation and increase lesion size.	[71,72]
Cytokine therapy	FGF1 injection Neuroregulin-1 Nerve growth factor	Increase of anti-inflammatory microglia Inhibition of microglia proliferation	Improved SCI recovery, but drug delivery should be enhanced	[74,75]
Stem cell transplantation	Human neural stem cells Mesenchymal stem cells	Inhibit microglial proliferation, migration, and phagocytosis	Showed capacity to enhance damage repair Novel materials and targeted delivery methods to optimize the outcome are needed.	[79,80]
Extracellular vesicles and nanoparticles	Neural stem cell-derived microvesicles Mesenchymal stem cells-derived microvesicles	Reduce microglial activation	Promising cell-free therapy Future research should optimize the efficacy using new materials and delivery methods.	[82,83,84]
MicroRNAs	miR-873a-5p miR-133b miR-124	Indirect microglia modulation via cytokine release	Showed potential in attenuating inflammation, oxidative stress, and apoptosis	[85,86,87]
Gut-brain axis	Different mouse models with limited bacterial colonization or complete microbiome depletion	Reduce microglia activation	Dietary interventions and antibiotic treatments influence the gut-brain inflammation axis, particularly the microglia.	[89,90,91]
Neuroprotective agents	Antioxidants Bradykinin antagonists Neurotrophins Peptide hormones	Regulation of microglial migration and recruitment	Showed the possibility of modulating pathways involved in BSCB impermeability	[92,93,94]

In recent years, drug delivery systems across the BSCB have evolved to enhance therapeutic transfer to injury sites. Nanoparticles, known for their nano-size, drug encapsulation, sustained release, and biocompatibility, have been extensively used for this purpose. Phase I/II clinical trials using nanoparticle-drug conjugates targeting brain tumors through the blood-brain barrier (BBB) are underway, demonstrating the potential of nanoparticles in traversing barriers. Nanoparticles from biological (exosomes) and synthetic (lipids) sources have shown promise in improving motor functions and restoring tight junctions to attenuate BSCB leakage post SCI [96]. Various types of nanoparticles, including metals, polymers, and lipids, have been explored as tracers and drug delivery systems in SCI, showing significant reductions in inflammatory factors and enhancement of neuronal regeneration [97]. A promising example of using nanosystems to deliver targeting drugs is the MyloGami approach [98]. In their study, Zhu et al. proposed a β-glucan-coated DNA origami loaded with topotecan, a topoisomerase 1 inhibitor (TOP1), for the myeloid-specific TOP1 inhibition and significant suppression of the microglial inflammatory response [98]. While nanomaterials have limitations such as toxicity and systemic clearance ambiguity, biological nanoparticles like exosomes warrant assessment as drug delivery carriers [99]. Additionally, the anatomical particularities of the BSCB (compared to the BBB) increase the degree of penetrability of nanoparticles, amplifying their benefits. These types of carriers are crucial to targeting smaller, deeper vascular repair in the spinal cord, particularly in grey matter, considering its heightened vulnerability after SCI [100]. However, existing studies are limited by short-term investigations, underscoring the need for long-term monitoring to understand BSCB disruption dynamics after injury fully.

## 5. Future Perspective and Challenges

To ensure the maximum effect of SCI treatments, a delicate balance must be maintained between the timing of interventions, individual variability, and the severity of the injury. Disparities between preclinical and clinical trial results, a lack of funding for scientific research, and the impossibility of targeting multiple pathophysiological pathways altered in SCI with a single drug are among the obstacles that remain even as research advances and new medications are investigated [101]. However, comprehending the complex mechanics of SCI and developing medicines that can target several pathways at once are promising directions. Furthermore, combining biological tissue engineering or cell transplantation techniques with drug therapy provides a comprehensive approach to treating SCI. Despite worries about unfavorable reactions to cell transplantation, the safety profile seems generally good, and most adverse effects are tolerable. There is growing evidence of its efficacy since numerous patients report improved motor and sensory abilities after a transplant [102]. Yet, there are insufficient large-scale trials, which calls for a more thorough investigation of the variables influencing treatment efficacy, the optimal time and dosage for cell transplantation, and the potential of combining other therapies. To broaden the range of possible treatments for SCI, it is also possible to assess novel cell types, such as multilineage-differentiating stress-enduring (MUSE) cells.

In recent years, exosomes have emerged as promising agents for SCI treatment. These functional extracellular vesicles, secreted by various cell types, offer a means of intercellular communication and hold significant therapeutic potential. Exosomes derived from various kinds of cells, including mesenchymal stem cells (MSCs), have been extensively studied for their reparative effects in SCI [103]. With studies showing MSC-derived exosomes attenuating apoptosis and inflammation, promoting functional recovery, and protecting the BSCB, the current direction in exosome research focuses on enhancing their therapeutic efficacy. Several tactics, such as loading the exosomes with specific nucleic acids or combining them with tissue engineering materials, such as peptide-modified hydrogels, are employed to improve sustained release and microenvironmental modulation.

Despite the robust preclinical data, significant obstacles remain in the clinical translation of exosome-based treatments for SCI. Clinical trial preparations must include standardizing exosome components, primarily derived from MSCs, and optimizing delivery strategies to guarantee targeted distribution to the damaged site. Exosomes can also be designed to modulate microglia activation or to target other pathological processes closely connected to microglial aberrant behavior in SCI. By resolving these issues, we may effectively use exosomes to treat spinal cord injury and get closer to clinical implementation.

Another promising direction comprises tissue engineering strategies, integrating cell biology, materials science, and molecular biology, offering promising avenues for repairing SCI [104]. These approaches involve the combination of various cells, factors, drugs, and materials to address multiple aspects of SCI repair. The main mechanisms include microenvironment modulation, including direct and indirect microglia modulation, promoting intrinsic regeneration, and bridging tissue gaps. Currently, scaffolds loaded with cells and cellular factors show the most promise. However, significant research is required to optimize scaffold selection, improve evaluation techniques, and conduct well-designed clinical trials for effective translation to clinical practice. Advancements in technologies like 3D printing hold the potential for creating more precise scaffolds to enhance anatomical structure and functional recovery.

Cell reprogramming technologies offer promising avenues for acquiring new neurons after SCI. Astrocytes, fibroblasts, and neuron-glia antigen 2-expressing (NG2) glia have been successfully reprogrammed into neurons using various transcription factors [105]. SOX2, Ascl1, and Neurog2 are among the critical factors involved in this process. Studies have demonstrated the potential of these reprogrammed neurons to promote neurogenesis and improve functional recovery in SCI models [106]. Microglia can be another interesting cell for reprogramming, with the modulation of its anti-inflammatory phenotype as a potential research direction. However, challenges remain, such as the low efficiency of reprogramming, safety concerns regarding gene therapy, and the need for further characterization of the reprogrammed neurons and their effects on the microenvironment.

Although reprogramming methods for treating SCI have not yet been the subject of clinical trials, research efforts have focused on assessing safety and efficacy in preclinical models. Despite promising results, concerns regarding the effect on the microenvironment and the functional properties of reprogrammed neurons still need to be resolved. Additionally, there is potential to overcome present limitations and promote the integration of these technologies into clinical practice by developing safer and more effective reprogramming techniques, such as using small compounds or CRISPR/Cas9 gene editing.

Lastly, the optimal timing and combination of the experimental therapeutic approaches remain the two still unanswered questions, which should also be resolved in the near future. While there is currently no consensus on the precise time to initiate experimental therapies, the variability of microglia phenotype is the main factor in choosing a therapy that modulates neuroinflammation or supports spinal cord repair/regeneration. Regarding the possibility of combining different therapeutic approaches, the first principle would be the use of therapies targeting different molecular pathways that might lead to an enhanced/synergic effect. Another essential aspect is related to the potential adverse effects, combining drugs carrying the risk of exacerbating unpleasant reactions. Finally, more research needs to be conducted to evaluate and compare the benefits and risks of microglia-targeting therapeutic combinations.

## 6. Conclusions

SCIs lead to debilitating and lifelong neurological impairments in an increasing number of young people worldwide. Microglia play a critical role in both inflammation and repair processes following SCI. Their activation is triggered by various factors such as ischemia, anoxia, and damage-associated molecular patterns. It exhibits distinct phenotypes and functions throughout different stages of injury and repair. In the acute phase, they release anti-inflammatory factors, recruit peripheral cells, and remove damaged tissues, but uncontrolled immune reactions can exacerbate the damage. In contrast, during chronic repair, microglia primarily limit inflammation and promote glial scarring, which may hinder neuronal regeneration and lead to chronic inflammation and damage.

Numerous therapeutic strategies targeting microglia have recently been proposed as adjuvant treatments in SCI. This heterogeneous group includes therapies based on CSF1R inhibitors, cytokines modulation, stem cells, microRNAs, and nanoparticles. However, achieving a precise balance of pro- and anti-inflammatory molecule secretion during different stages of SCI is crucial for optimal outcomes. Modulating microglial polarization through different mechanisms remains challenging for resolving inflammation and enhancing recovery post-SCI.

Despite extensive research in the field, there is still a mandatory need to better understand microglial activation and functions for developing targeted therapies to alleviate secondary damage and promote repair in SCI. Future research should focus on adjusting the proportion of different microglial phenotypes to achieve better functional recovery. While much of the evidence comes from animal studies, ongoing clinical trials promise to translate these findings into improved outcomes for SCI patients in the near future.

## Figures and Tables

**Table 1:** Microglia as the critical factor modulating multiple processes in spinal cord injury.

Process	Alterations in SCI	Microglia Impact/Changes	Most Relevant References
Pro-/anti-inflammatory aspects	Increased production of inflammatory cytokines (TNF-, IL-1), chemokines, prostaglandins, and reactive oxygen	Microglia activation (morphological + phenotype change)	[44,45,46]
Phagocytosis	Increased turnover of injured axons	Increased phagocytic activity Very efficient debris processing	[47,48]
Neurodegeneration	Glial scar formation inhibits neuronal regeneration	Production of cytotoxic cytokines Neurotransmission modulation	[50,51]
Astrocyte functions	Increased astrocyte proliferation and activation	Secretion of IL-1α, TNF, and C1q induces A1 astrocyte Secretion of FGF induces A2 astrocyte	[54,55,56]
Other glial cell (oligodendrocytes, progenitor cells) functions	Alterations in oligodendrocyte survival Influence of oligodendrocyte remyelination	Secretion of TNF, NO, and complement Modulation of NF-κB signaling pathway	[63,64,65]
BSCB permeability	Increased BSCB permeability, leukocyte infiltration, and angiogenesis	Production of reactive oxygen species, IL-1β and TNF-α Downregulation of TJ proteins	[57,58,59,60,61,62]

## Data Availability

The data presented in this study are available on request from the corresponding author.

## Abbreviations

Ang1	angiopoietin-1
BBB	blood-brain barrier
BDNF	brain-derived neurotrophic factor
bFGF	basic fibroblast growth factor
BSCB	blood-spinal cord barrier
CNS	central nervous system
EV	extracellular vesicles
GDNF	glial-derived neurotrophic factor
IL	interleukin
MMP	matrix metalloproteinase
MSC	mesenchymal stem cells
OPC	oligodendrocyte progenitor cells
SCI	spinal cord injury
TJ	tight junction
TNF	tumor necrosis factor
